# Henri Michaux's program for the psychedelic humanities

**DOI:** 10.3389/fpsyg.2023.1152896

**Published:** 2023-05-16

**Authors:** Oliver Davis

**Affiliations:** School of Modern Languages and Cultures, University of Warwick, Coventry, United Kingdom

**Keywords:** Michaux, psychedelic, mescaline, psilocybin, esthetics, politics, autoheteronomy, anthropotechnics

## Abstract

This article presents an analytical reading of the extraordinarily rich cultural production around drugs by the 20th-century French poet, writer, critic, and visual artist Michaux (1899–1984). Over about a decade, from the mid-1950's, the otherwise habitually sober Michaux wrote five books, included within which were dozens of drawings, and made one half-hour film, charting his adventures as an initially reluctant yet persistent psychonaut, principally with mescaline, but also with psilocybin, LSD, and cannabis. This has rightly been described as one of the most creative cultural explorations of mescaline. It is more extensive, texturally complex, and esthetically demanding than Aldous Huxley's far better known near-contemporaneous published work on psychedelics in English, which is well-known within and arguably foundational for psychedelic studies. Yet, this very complexity, as well as the national-linguistic context of its articulation—there was no mass psychedelic counterculture in France—have limited wider engagement with it. I argue that Michaux's esthetic reconstruction of psychedelics' effects on his creative brain can be read as a “program” for the emerging field of the psychedelic humanities and that it makes a substantial contribution, which I sketch in outline here, to the following of core concerns: (1) the role of psychedelics in enhancing “creativity”; (2) conceptualization of the politics of psychedelics; and (3) the meaning and value of psychedelic mysticism. I aim to show that Michaux's work on drugs has much to contribute to the cultural understanding of psychedelics today and accordingly that this unjustly neglected classic of French—and global—drug culture deserves to be far better known.

## 1. Introduction

### 1.1. Background

The five books of writing, which also contain dozens of drawings, and the half-hour film, which issued from Henri Michaux's psychonautic self-experimentation with mescaline during the 1950's and 1960's (Michaux, 1956, 1957, 1959, 1961, 1966; Duvivier, 1963), have been described as the century's “most sustained and creative” engagement with this particular psychedelic (Jay, 2019; p. 211).^1^ They also have more to say about cannabis, psilocybin, and LSD, although their main focus, like mine in this article, is on mescaline. In part because of their textural complexity within and across three distinct media (writing, drawing, and film) and the extremes of ambivalence they record about the various experiences, as well as the national-linguistic context of their articulation—there was no mass psychedelic counterculture in France—they have not been read nearly as widely or attentively as they deserve. Put more positively, the resurgence of cultural interest in psychedelics, which has accompanied their “renaissance” (Sessa, 2012) within psychiatry, allows new questions to be posed about this hitherto underappreciated material.^2^

For largely contingent reasons of translation, the uptake of Michaux's work during the American psychedelic counterculture was more limited than it might have been: the first but also the most skeptical book of the series, *Misérable Miracle* (Michaux, 1956), was published in English translation early enough, by the San Franciscan countercultural publisher City Lights, in 1963 (Michaux, 1963a).^3^ The fourth, *Connaissance par les gouffres* (Michaux, 1961), also appeared in translation in the same year, as *Light Through Darkness* (Michaux, 1963b), but of all the books in the series, this is the one most constrained by the psychotomimetic paradigm. However, the second, decidedly more enthusiastic, upbeat, and in some respects most mystical book, *L'Infini turbulent* (Michaux, 1957)—the one most attuned to the temperament of the American psychedelic counterculture—did not appear in English until much later, in the mid-1970's (Michaux, 1975). While Michaux's influence on the counterculture was limited, he was well acquainted with several of its key texts, concerns, and people: for example, in *L'Infini turbulent* (Michaux, 2001,[1957]; p. 814), he echoed Huxley's discussion (Huxley, 2004 [1954]; p. 34–5) of the *Bardo Thödol* (“*Tibetan Book of the Dead*”), and he met Allen Ginsberg in Paris in 1958 (Ginsberg, 1995; Morgan, 2006; p. 274; p. 346), gave him a signed copy of *L'Infini turbulent* (Michaux, 1957), and the pair subsequently corresponded (Martin, 2003; p. 549).^4^

Alongside these contingencies of translation, which I believe largely explain the limited uptake of Michaux's work by the American counterculture, it is worth noting that in key respects Michaux's general outlook on psychedelics was narrower and somewhat more uptight: he only took laboratory-synthesized mescaline rather than peyote and he flaunts this decision by giving the structural formula of mescaline hydrochloride as one of the two epigraphs to *Misérable Miracle* (Michaux, 1956, 2001; p. 618). Moreover, despite having written about his travels in Central America earlier in his life (Michaux, 1929) and being familiar with some indigenous uses of peyote - he mentions the Huichol and the Tarahumara, as well as some of the geometric features of Aztec art and architecture on several occasions, for example - he couches his own interest resolutely in terms of Western biomedicine. He does not discuss the role of peyote in “postcolonial healing” (Calabrese, 2013) by the Native American Church, nor the significant stewardship of the peyote experience by that institution, and it is possible that he was unaware of it. As the Beat poet, essayist, and stalwart of the American counterculture Michael McClure noted, “A mescaline high is not a peyote high” (McClure, 1966; p. 42), and Michaux was aware that he was exposing himself to just one of the several psychedelic alkaloids in the peyote cactus.^5^ This preference for synthetic mescaline and relative indifference to indigenous practices also placed Michaux at variance with two significant earlier French authors on peyote: pharmacist, entrepreneur, and occultist Alexandre Rouhier, who dedicated his classic study to “THE SPIRIT OF CUAUHTEMOC” (Rouhier, 1927; p. iii, capitalization in original) and Antonin Artaud (Artaud, 1945).^6^ I discuss the political ramifications of these choices of Michaux's in Section 3 below.

In France, there was little interest in Michaux's drug books initially, with the first edition (Michaux, 1956) selling only around 250 copies. He nevertheless acquired a certain notoriety for his experiments with psychedelics and a planned public screening of his film was banned in November 1968 (Michaux, 2004; p. 1540), though this must also be attributed to the generalized repressive crackdown following the protests of Mai '68. Prohibitionist drug law in France, as in other countries, significantly tightened with regard to mescaline during the period in which Michaux experimented with the substance and its immediate aftermath: while his particular use of mescaline was, in the strictest of senses, legal when he began his experimentation in the mid-1950's, it was no longer legal with the passing of the 1970 law explicitly prohibiting its use and whether his writings about drugs fell foul of other prohibitionist provisions is a moot point.^7^

The remainder of this Introduction will outline the settings in which Michaux took psychedelics, his typical doses, his aim or “set,” and his familiarity with contemporaneous biomedical research on psychedelics, in which the psychotomimetic paradigm was dominant, before the substantive sections of the article argue for a reading of his work as a programmatic template for the psychedelic humanities.

### 1.2. Setting

Most of Michaux's drug experiences took place at home, in his flat on the Rue Séguier in central Paris. They began on 2 January 1955 (Pic, 2014; p. 9), when Michaux, like the century, was in his mid-fifties and already a very well established and well connected, yet by repute, rather an aloof writer, poet, and visual artist.^8^ The first experience took place in the company of some five friends or acquaintances and his housekeeper; the mescaline was probably supplied by psychiatrist Dr. Julian de Ajuriaguerra (Ouvry-Vial, 1989; p. 200; Martin, 2003; p. 518). Most of the subsequent mescaline experiences were conducted with others present, or in an adjacent room, although Michaux occasionally took the drug alone, but only after having notified a friend by telephone, who would ring back 3 h later to check on him (Martin, 2003; p. 520).

Michaux's first experience of psilocybin (recounted in Michaux, 1961) took place in 1958 in the very different setting of the Sainte-Anne psychiatric hospital in Paris, as part of a clinical study (Delay et al., 1959) overseen by the charismatic director of its Clinic of Mental Illnesses and Illnesses of the Brain and also Chair of Psychiatry in the Paris Faculty of Medicine, Jean Delay. Delay was a friend of Michaux's and is generally remembered for his role in the discovery of the antipsychotic effects of chlorpromazine in 1952 (Thuillier, 1999; Healy, 2004; p. 88), but he had also been leading clinical studies with mescaline from the mid-1940's (Delay and Gérard, 1948; Dassonneville, 2021; p. 89), following on from similar research conducted at the same institution on mescaline, by others, in the preceding decade (Dassonneville, 2021; p. 80). Delay also worked with LSD throughout the 1950's (Thuillier, 1999; Dubus, 2022). His experiments with psychedelics on psychiatric in-patients at Sainte-Anne had no regard for the role of “setting” or “set” and, even by the psychiatric standards of the day, were in some cases remarkable for their cruelty and obliviousness to patient consent (Dubus, 2022). Nevertheless, in the decidedly different quality distinguishing Michaux's two narrated experiences with psilocybin, recounted sequentially in *Connaissance par les gouffres* (Michaux, 2004; p. 16-36), the first at the hospital under clinical observation (but none of the other constraints imposed on in-patient subjects) and the second at home, the significance of “setting” was already registering in France in the early 1960's, despite Delay's indifference yet on the literary fringes of his entourage, in Michaux's work in the psychedelic humanities.

### 1.3. Dose, substance, and aim or “set”

Generally, Michaux took mescaline hydrochloride in doses under 0.3 g, though on one occasion, he took 0.6 g, “six times the sufficient dose for me,” owing to “an error of calculation” (Michaux, 2001; p. 723)—a “strange error” indeed (Blanchot, 1966; p. 83) given that it came in capsules of 0.1 g—and had a very intense “bad trip,” only calmed by two home visits from his sympathetic doctor later in the day.^9^ In other words, Michaux preferred to consume mescaline in quantities just above the typical threshold dose (his “sufficient” dose) of 0.1 g and rise to an intermediate dose of 0.3 g (Erowid, 2015), presumably so that he could still steer the trip and “Observe the derangements [*les dérèglements*], the erroneous connections in thought, the errors of the thinking instrument, now upskittled, and the illusions of the human being who possesses this fragile thinking instrument.” (Michaux, 2001; p. 770). That this constitutes a relatively open and exploratory “set” is a point to which I return in Section 2 below.

### 1.4. Contemporaneous biomedical research on psychedelics and literary precursors

Several years before commencing his self-experiments with mescaline, Michaux began reading widely in contemporaneous psychiatric research on psychedelics (Martin, 2003; p. 514) and the drug works taken together contain around 80 note references to scientific publications (Halpern, 1998; p. 19). Six of these are elogios references to Delay, who thereby figures as Michaux's “scientist-double” (Halpern, 1998; p. 19), a relationship somewhat reminiscent of the Anglophone writer-psychiatrist dyad of Aldous Huxley and Humphry Osmond (Bruchez, 2007; Dyck, 2008). Delay returned the favor by concluding an article on LSD with reference to Michaux's evocation of the “miserable miracles” of a “neighboring experience,” in other words with mescaline (Delay and Benda, 1958; p. 342), and by prefacing his film (Duvivier, 1963), to some extent thereby testifying to its scientific interest. Michaux was also on friendly terms with pioneering mycologist Roger Heim, beginning a correspondence of some 20 letters with him in 1958; Heim supplied Michaux with synthetic psilocybin and psychedelic mushrooms (Michaux, 2004: xii, 1485). Michaux also read older scientific works on peyote and mescaline by Ellis (1898), Rouhier (1927), and Lewin (1928) (Martin, 2003; p. 514-5). He was well versed too in more literary engagements with psychoactive drugs, notably Thomas De Quincey's *Confessions*, a foundational text for psychonautic drug-writing (Partridge, 2018), work by Baudelaire (1860) on cannabis and opium, and the more proximate engagements, with mescaline, by Jean-Paul Sartre in the 1930's [Sartre's own account was published posthumously in Sartre (2010), but had circulated in unpublished form and is mentioned in Merleau-Ponty (1942, 1945); see also Dassonneville (2021) and Farrell (2021)] and by Artaud in the 1940's (Artaud, 1945).

### 1.5. The psychotomimetic paradigm

As well as Michaux's likely supplier, Ajuriaguerra was the co-author of the first monograph on Michaux's drug writing (Ajuriaguerra and Jaeggi, 1963), which construed the experiences largely in terms of the “model psychoses” or “psychotomimetic” paradigm (Swanson, 2018) dominant in psychiatric research on psychedelics during the 1950's but dating back, as a paradigm for the effects of psychoactive drugs more widely, to the mid-nineteenth-century work of Jacques-Joseph Moreau (“de Tours”) on cannabis, among other substances (Foucault, 2003; p. 280-84). Ajuriaguerra and his co-author's reliance on this paradigm are unsurprising given its dominance in the day and the way Michaux indulges in largely speculative comparisons between his experience and what he observed of the behavior of patients at the Sainte-Anne and other psychiatric hospitals, where he was seemingly permitted fairly free access to wander the wards making amateur observations. Nevertheless, adherence to this now obsolete paradigm is by no means complete, or sustained evenly, in the works; as the substantive part of this article now aims to show, they survive the obsolescence of that paradigm.

## 2. The psychedelic enhancement of “creativity”

Although the main focus of research in the “psychedelic renaissance” to date has been on the medical and therapeutic potential of psychedelics for treating diagnosed mental health disorders, the question of whether psychedelics can enhance creative thinking more widely, and if so how, remains open (Sessa, 2008) and has recently been posed in relation to scientific creativity specifically (Gandy et al., 2022). “Creativity” is a particular focus in research on microdosing, whether in the form of analyses of self-reports (Anderson et al., 2019; Petranker et al., 2020) or an ongoing RCT study (Murphy et al., 2021), the results of which are eagerly awaited given suspicions that self-reported benefits of microdosing might be placebo effects.^10^

“Creativity” was a preoccupation of pre-prohibition research on psychedelics, as is indicated by the name of Al Hubbard's Commission for the Study of Creative Imagination, established in 1955, to extend the study of these substances beyond medical use. “Creativity” was a particular focus of research on the West Coast of the United States, the cradle of the often forgotten “technophilic” counterculture (Turner, 2006), notably undertaken under the auspices of Willis Harman and Myron Stolaroff's International Foundation for Advanced Study in Menlo Park, California, on the fringes of Stanford University; some of this research involved, as participants, Douglas Engelbart and other pioneering computer developers from his Augmented Human Intellect Research Center at the Stanford Research Institute (Markoff, 2005; p. 67). Rather than research involving art or artists, “creativity” in this strand of pre-prohibition research was largely understood as the resourceful and successful solution of technical problems, in a manner akin to the popular-psychological notion of “lateral thinking” (De Bono, 1970).^11^

The most significant of these pre-prohibition “creativity” experiments, using mescaline (Harman et al., 1966), involved as participants engineers, architects, and scientists, each of whom was asked to bring to the study one unresolved technical problem, on which they had been working for some time and had become “stuck.” The study concluded that above-threshold but moderate doses of the psychedelic, administered under conditions of “appropriate expectancy” (Harman et al., 1966; p. 216), enabled “enhanced ability to recognize patterns” (219), “deautomatization” (221), “[h]igh movitation to obtain closure; an appetite for elegance” (224), and the “[c]apacity to visualize the completed solution in its entirety” (224).^12^ For successful “creative” problem-solving by humans, this study suggested that technical, motivational, perceptual, and cognitive aptitudes are all required. One of the abovementioned microdosing studies (Anderson et al., 2019), based on a grounded theory analysis of self-reports by microdosers, concluded that “creativity” came third in a list of benefits ascribed to the practice, after “improved mood” and “improved focus” and before “self-efficacy” and “improved energy.” Most of these are recognizably similar, despite the different language, to many of the benefits registered in the landmark 1966 macrodose study (Harman et al., 1966), which made a point of trying to focus narrowly on the technical problems and aptitudes required to resolve them and preparing participants pre-administration to avoid addressing problems of a personal nature during the trip. For professional problem-solvers, it is reasonable to suppose that “improved mood” would also follow from a successful resolution of the problem. My point is not to indulge in idle speculation about the relationship between two very different types of studies conducted under very different conditions some half a century apart but rather to observe that, conceptually speaking, work on the psychedelic enhancement of creativity tends to look for a cluster or multiplicity of aptitudes in which the perceptual, cognitive, and motivational are closely intertwined. In so far as scientists draw their understanding of “creativity” from the surrounding culture, in a process of abstraction and reconstruction that is explicitly built into the grounded theory methodology of the microdosing study (Anderson et al., 2019), their understanding and what they look for empirically will to some extent reflect the fuzziness, or clustered multiplicity, which characterizes this concept in ordinary usage.^13^

As Mason et al. (2021) note, “Creativity is an essential cognitive ability linked to all areas of our everyday life, allowing us to adapt to an ever-changing environment and come up with ways to solve problems.” Creativity can in this sense be considered an essential human aptitude. Nevertheless, ordinary cultural usage does also recognize one particular activity more especially concerned with creativity than others: the making of art. Even those who concur with Joseph Beuys saying “Everyone is an artist” (Michaud, 1988; p. 36) cannot escape the fact that in the current social division of labor, those we call artists are especially concerned with creativity. It is plausible to suppose that creative artists who have experimented with psychedelics might have something substantial to contribute to the cultural conversation from which scientific conceptualizations of elements within the creativity cluster are drawn and it is in this spirit that I turn to Michaux.^14^ What can he add? His initial verdict (Michaux, 1956) on the impact of mescaline on his creative imagination is entirely negative: “Mescaline diminishes the imagination. It castrates the image, desensualizes it. It makes images that are 100% pure, laboratory grade. […] Thus it is the enemy of poetry, of meditation and above all of mystery.” (Michaux, 2001; p. 674). Furthermore, he complains of the drug's aftereffects: “Two weeks after the last experiment I was still unable to write except repetitively, in the most banal of ways; this is largely due to a lack of (natural) images […]. Even in conversation, although more garrulous, less restrained, I had become a pauper as far as images are concerned.” (Michaux, 2001; p. 674). The mescaline visuals are dismissed as a “tacky retinal circus” (Michaux, 2001; p. 632) and although he does not use the word, Michaux seems to object, in effect, to their *kitsch*: they are “*shockingly like advertisements*” (Michaux, 2001; p. 624, italics original). Their garish colors, their insistence, and their schematic or abstract quality, which detaches them from the realm of sensuous experience, make them “the enemy” of “poetry,” in Michaux's initial judgment. We may not share the rather conventional assumptions implicit in this verdict about what making (good) art, or conversation, involves—the production of sensuously rich “images”—but if we compare Michaux's experience with the findings of the 1966 creativity study discussed in the preceding paragraph (Harman et al., 1966), substituting “images” for “solutions,” then it seems that his early experiences of mescaline ran counter to those of the engineers and scientists enrolled on that study and resulted, so to speak, in a lowering of the rate of artistic production.

In his early encounters with the drug, even before the fourth experiment's dosing “error,” mescaline seems to frustrate Michaux's capacity to produce creative work. The mescaline experiences are initially an unwelcome disruption to his settled ways of working creatively. Yet, they lead eventually to a body of work which is remarkable not for the “images” it contains, less for its “content,” and more for how, by working through the adversity of that disruption, Michaux undertakes a formal and textural esthetic reconstitution of the pullulating profusion—the sense of sprouting and generative multiplicity, of chaotic creative potentiality—which characterizes his experience. The first two books in the series (Michaux, 1956, 1957) adopt a novel textual practice whereby sparse marginal annotations in italics sit alongside the main body of the text, effectively introducing two columns of text on each page.^15^ Michaux commented in the preface to *Misérable Miracle* (Michaux, 1956): “In this book the margin occupied more by shortcuts than titles expresses very insufficiently the overlappings [*les chevauchements*], a phenomenon always present with Mescaline […]. No other ‘devices' have been used. Too many would have been needed.” (Michaux, 2001; p. 620) A double text is thereby created, which is, as it were, doubled again by the inclusion of the drawings in the first three of the five books (Michaux, 1956, 1957, 1959), and the writing and drawing are in turn doubled by the filmic representation (Duvivier, 1963). This textual doubling, or multiplication—this proliferation of the text into text plus paratext, complicated in turn by proliferation across media into drawings and film—can be understood as an attempt to go some way toward expressing formally the pullulating multiplicity characteristic of Michaux's mescaline experience and the tendency for elements within that experience to overlap, influence, and interact with and impinge on one another—and to share that experience with his audience in the way that it obliges them to read and see differently.^16^ The iterative formal reduplication of the text can be understood as the expression, in terms more of form than content, of his experience of pullulating multiplicity and multiplication under mescaline: “Pullulation! Pullulation everywhere! Pullulation from which there is no exit. Space full to overflowing, space of gestation, space of transformation and multiplication; teeming space, which, even if it were only an illusion, would give better account than ordinary sight of what the Cosmos is.” (Michaux, 2001; p. 679).

Considering Michaux's work alongside the early creativity study, I surmise that mescaline provoked a radical “deautomatization” (Harman et al., 1966; p. 221) of his creative practice, creating an initially unwelcome disruption of his settled ways of working but which ultimately enabled, through his deliberate and repeated practicing against this new coefficient of adversity, a type of creativity quite unlike the production of sensuous images or literary “content” and much more concerned with inventions in texture and form. The “solutions” mescaline offers Michaux lie not in ready-made images that might be captured from its visuals and reproduced citationally on the page but rather in the way in which the psychedelic upskittled his well-established ways of working and enabled or forced him to develop an innovative and expansive new practice of the form. In an illuminating counterpoint to biomedical research on the psychedelic enhancement of creativity, Michaux's work suggests that psychedelics do not always enhance creativity simply by increasing output within existing forms and frameworks. They sometimes first dismantle those forms and frameworks: they disassemble the production line, so to speak, and deautomatize the production. They thereby clear a space in which the subject can reconfigure the terms of representation, remaking the forms, tools, and techniques of representing: when enhanced by psychedelics, creativity can also involve destruction and stoppage. I would speculate that the capacity to rebuild new forms and frameworks depends in part on training and discipline, and the enhancement in creativity on the will to continue “practicing” with the creatively destructive psychedelic technique. I return to the significance of such anthropotechnical practice in my conclusion. Evidently, Michaux's experience of mescaline was far more disruptive and, at least initially, chaotic than that reported by the engineers, architects, and other macrodosed tech workers [in Harman et al. (1966)]. Why might this be? Perhaps because he approached the drug with a relatively open set, as noted above (Section 1.3): he did not have a specific problem to solve, nor do we have any evidence that he was depressed, or “stuck,” personally or professionally, nor that he was prone to psychosis. Under these relatively open conditions of exploration—starting with the relatively open set characteristic of the psychonaut—he seems to have had a more radical experience of creativity as an original pullulating or potentiating chaos before the emergence of order and form.

Michaux's early descriptions of mescaline's effects often characterize the drug as a mechanism operating inside him, heteronomously. He begins to realize that it will work upon any thought he feeds it: observing to himself that the “himalaya” mountains he visualizes are “immense,” the two-letter m's in this adjective suddenly shoot off upwards and become “arches for unthinkable and baroque cathedrals” (Michaux, 2001; p. 624).^17^ As he begins to discover that mescaline enhances his capacity for self-suggestion, he resolves to try not to think of anything: “Let's not give one idea, not one item, to this crazy mechanism. But already the machine had begun moving again at one hundred images per minute.” (624) The mechanistic quality he attributes here to the drug could perhaps be understood as his experiencing, under its influence, the limitations of his own mechanistic self-conception, in particular, as this involves unhelpful capitalist-productivist assumptions about what it means to be productive as a creative artist, or even as an imitative reactualization from his reading of earlier trip reports [notably Rouhier (1927); p. 252].

The abstracting effect which he attributes to the drug leads, in his esthetic reconstitution of the experience, to an explosive experimentation with form and medium by way of textural complication and transmedial expansion. Most significant and consequential is the fourth experiment [in Michaux (1956)], which begins with the abovementioned dosing “error” such that, unusually, he consumes a “heavy” dose of 0.6 g (Erowid, 2015). Michaux experiences becoming letters and a line: “Large Z's are passing within me (zebra-stripes-vibrations-zigzags?). Then it is broken S's, or then again, perhaps halves of them, incomplete O's” (Michaux, 2001; p. 733); “To have become a line was catastrophic, but it was also, if this is possible, all the more unexpected and prodigious.” (Michaux, 2001; p. 738). In other words, Michaux experiences becoming one with the very matter of his creative activity, letters, and lines, in a psychedelically enabled immersive expression of the renewed focus on medium and form often thought to characterize esthetic modernism. Michaux's experience bears some resemblance to the self-report by biochemist Kary Mullis on his discovery of how to automate the polymerase chain reaction, a discovery he was convinced had been enabled by self-experimentation with LSD: “I was down there with the molecules” [cited in Doyle (2011); p. 193]. In both Michaux's and Mullis's cases, the psychedelic trip enables a radical perspectival shift, an immersive empathic-projective visualization of the scenario at a microscopic level with a high degree of intensity: “I was living intensely in microperception, among the microsignals” (Michaux, 2001; p. 997). It is in the cognitive yield enabled by this engrossing shift of perspective to the microperceptual level that the successful “creative” problem-solving documented in the 1966 creativity study (Harman et al., 1966) might best be understood and explicated: the mind becomes a much more sensitive and more incisive instrument, reattuned to the basic elements of the problem, visualizing them at the microperceptual level, and capable of remaking the forms and frameworks of its understanding around those elements.

## 3. The politics of psychedelics

As well as offering insight into the way psychedelics enhance creativity, the shift to the microperceptual documented in Michaux's drug works sheds new light on the increasingly vexed question of the politics of psychedelics. For the historically contingent reason of their entanglement with a left-leaning counterculture, it has often been assumed that psychedelics are conducive to greater openness to other people and cultures, as well as the profound realization of human interconnectedness with other species and the natural environment, for example, in the suggestion that psychedelics might be “ecodelics” (Doyle, 2011). This way of thinking has been reflected in the theorization of Acid Communism by Mark Fisher (Stamm, 2019) and Psychedelic Socialism by Jeremy Gilbert (Gilbert, 2017), as well as some outlying biomedical research (Nour et al., 2017; Lyons and Carhart-Harris, 2018). However, a longstanding line of skepticism about such claims, which dates back to the 1970's (Felton, 1972), is now gaining ground in the psychedelic humanities, as scholars point to the penchant for psychedelics among some right-wing ideologues historically (Piper, 2015), the wider phenomenon of “Rightist Psychedelia” (Langlitz, 2020) and the interest in these substances in some corners of the alt-right today, including Q-Anon and neo-Nazism (Pace and Devenot, 2021). Attempts to conceptualize the politics of psychedelics would thus appear to have reached an impasse: the substances seem to be conducive to either extreme, or a number of extreme positions, with research to date suggesting that the most we can say is that they eschew the centrist middle-ground of liberal democracy and that they are politically versatile, or “pluripotent” (Lonergan, 2021), and conducive to the entrenchment of any already held belief.

However, this impasse presupposes quite a conventional view of politics, which envisages the political in terms of already constituted macropolitical positions and the subjects who hold to them. There is another way of looking at politics, well-established in theorization of radical democracy, which offers a more promising approach better attuned to the way psychedelics function—and this is probably no accident of history. Advanced by Deleuze and Guattari (1984 [1972]; 1987 [1980]) and Guattari (2012), then recrafted by Jacques Rancière, this “molecular” perspective on politics focuses on the micropolitical processes by which macropolitical (“molar”) institutions, positions, and subjects who hold them come to be constituted.^18^ An exhaustive account of this approach is impossible within the constraints of the present article (see Davis, 2010; p. 74–100) but its merits are expressed succinctly in Michaux's comment on the microperceptual perspective which psychedelics enable: “Everything or almost everything is constituted, constituting and thus reconstituable.” (Michaux, 2004; p. 33) Reading through the lens of these “molecular” theorists of politics, Michaux's work suggests that the psychedelically trained mind's sensitivity to infraperceptual phenomena which remain below the level of ordinary awareness gives rise to a conviction that any constituted object of consciousness, including those shared culturally and politically, might be remade anew. This is not magical thinking but rather the subjective “molecular” ground of the indispensable political conviction that things could be otherwise.

If the political import of psychedelics is to show that every object of common political belief and every believing subject might be remade anew, as Michaux and the political thinkers he influenced suggest, little wonder that psychedelics seem to appeal more to thinkers of radical, extreme, or revolutionary politics and to alarm those who prefer the centrist middle-ground of consensual, representative-electoral liberal democracy. Does this mean, however, that psychedelics are entirely versatile in political terms and are unwedded to any particular form of politics? While their use in numerous indigenous cultures as agents of socio-cultural consolidation and reproduction is well-documented (Dobkin de Rios, 1990 [1984]), in a social setting that is already heterogeneous, it is highly unlikely that psychedelics could readily serve the same consolidating purpose. The very “wildness” (Langlitz, 2012; p. 131) of these substances—the difficulty of predicting and stabilizing their effects—militates against this. For psychedelics to function reliably in such a way would require that setting and set already be controlled so comprehensively as to make the political use of psychedelics redundant: if a regime already had control of its subjects' mindset and environment to such an extent, there would simply be no need to call on the amplificatory effects of psychedelics.^19^ I call this the *redundancy thesis*: it posits the redundancy of psychedelics for authoritarian macropolitical organization, whether right or left. Rather, psychedelics are interruptive, “molecular,” emancipatory political technologies of radical freedom and emergence which are far more likely to weaken established macropolitical structures than to consolidate them. This does not mean that they are politically redundant—far from it—or that the old consensus about psychedelics being conducive to left-leaning politics can be restored: to say they are “molecular” technologies of radical freedom is not necessary to align them with left-wing politics but it is to oppose them to organized political authoritarianism of any stripe and to calm mounting panic at the prospect they may stand set to usher in a fascist future. A new account is required of how the “molecular,” or micropolitical, activity of psychedelics and those groups who make use of them can transform macropolitical structures. Prominent in such an account will be many of the same aptitudes discussed in Section 2, under the enhancement of “creativity”: from a “molecular” perspective, creative problem-solving is not only an individual but also, fundamentally, a “transversally” intersubjective matter (Guattari, 2012); the methodological individualism of the psy-sciences and the individualization which therapies derived from their research produces, reflect, from a “molecular” perspective, arbitrarily anti-social decisions.^20^

In addition to inspiring a “molecular” approach to politics, of the type encountered later in theorizations of radical democracy, Michaux's work makes another contribution to the understanding of psychedelic politics by registering, resisting, and partially interpreting a tendency toward what might be called delusions of grandeur. “One is overcome by superlatives. One suffocates with superlatives. One would scream superlatives. One is immense and radiant with superlatives. One is thirsty and in great need of superlatives. The greatest and most extraordinary. One is insatiable. One lives superlatively.” (Michaux, 2001; p. 812). Michaux is wary of this propensity toward the superlative: “If I had given something of myself to this, it would certainly have led to megalomania. In sum, the strings of the megalomaniac were being given a sharp tug. A sharp and mechanical tug. So I didn't respond. […] Perhaps one day the ingestion of Mescaline and some other well-chosen drugs will be made compulsory at university level for future ‘leaders [*manieurs*]”' (Michaux, 2001; p. 693). Michaux lacked the messianic ambition of Timothy Leary; his advocacy for psychedelics was far more contorted, unwieldy, and ambivalent—for characteriological reasons, I would suggest more than to avoid the French legal prohibition on proselytizing for drugs (see n.7). Nevertheless, it is extraordinary that Michaux not only apprehends and resists the “maximomaniacal pressure” (Michaux, 2001; p. 812) he experiences in his encounter with mescaline but also envisages a future in which psychedelics will be put to technocratic use in the training of political leaders. That is, in a far more sinister vein than Leary's vision of the university in which psychedelics would eventually replace books as anthropotechnical devices for the fashioning of selves, Michaux speculates about a strongly hierarchized technocratic political future in which psychedelics will have become part of the curriculum for training an elite destined to govern by moving the masses with carefully administered doses of charisma.^21^

How can this distinctly authoritarian vision of the future be reconciled with my earlier claim that Michaux's drug writing inaugurates a “molecular” conception of politics according to which psychedelics tend to undo organized political authoritarianism? Like many artists and intellectuals preoccupied with their own creative activities, Michaux had what might be described as solipsistic or even, in his case, autistic tendencies: “*Evil is other people's rhythm*,” he wrote in 1949 (Michaux, 2001; p. 342, italics original). When he imagines the possible advantage which psychedelics might give to the leaders of a hierarchical technocratic state of the future, he, like some of today's oligarchs, pharma entrepreneurs, and “psychedelic pundits” (Devenot et al., 2022) and some of their critics, does not pause to consider that others with very different political viewpoints starting from much less privileged positions could also enjoy a similar benefit but to different ends. He does not imagine, but his readers can, the effect of such a boost in political self-belief on the undermotivated and quietly despairing multitudes who might lack the basic self-esteem and self-confidence which, according to philosopher Axel Honneth's recognitive account of autonomy, for example, are essential proto-political conditions for the exercise of this and other aspects of political agency: “molecular” conditions, even though Honneth does not use this term (Honneth and Anderson, 2005). There is good reason to believe that without quite being above the threshold at which they might be diagnosed as clinically depressed, a sizeable proportion of the world's downtrodden lack the motivational means and self-belief to engage in projects of individual or collective transformation: they are held captive by their situation, beaten down by economic hardship and social deprivation, caught up in flows of information, “guidance,” and “entertainment.” Reading against its grain, from the perspectives of radical democracy and recognitive theory outlined here, perforce briefly, Michaux's anticipation of a psychedelically assisted technocratic future suggests that there would be a considerable transformative benefit in the psychedelically assisted self-raising of their self-esteem and motivation by a despondent global majority, indeed that this would in effect consolidate the force of their political will.

There is one respect in which Michaux's drug works are politically problematic: as mentioned (Section 1.1), their orientation is resolutely toward Western technoscience and biomedicine and engages only very fleetingly with indigenous cultural practices. In this one respect, Michaux's approach is rather narrow and ignorant and I would not wish to suggest otherwise, though at least he is transparent about this orientation and, as the following section establishes, he is to some extent consistent or evenhanded in the sense that Christian mystical experience is also subordinated to scientific explanation and translated into secular naturalistic terms. Had he been questioned on this point, I can imagine him responding along these lines: however significant indigenous practices may be, like it or not, Western technoscience is now the hegemonic paradigm and unless indigenous experiences can be translated into its terms they are destined to remain of largely antiquarian interest. They can certainly be “recognized,” as many scholars in the psychedelic humanities tirelessly demand, but whether much follows concretely from earnestly felt rhetorical gestures in this direction is a decidedly moot point. Of course, Michaux's approach contrasts markedly with that of some scholars in the psychedelic humanities, who wish to envision a future that “respects the lineages of the knowledges that are essentially and not accidentally bundled with these plants—Indigenous and counterculture wisdoms” (Devenot et al., 2022). However, Michaux is less interested in “plant medicines” than in synthetic chemical forms and, like it or not, the field of psychedelics is now very much wider than that of plant medicines and cannot be reduced to plant medicines. Even if one agrees with the sentiment of these authors that indigenous uses constitute an invaluable archive of techniques, as I do, a treasury of techniques sometimes at variance with Western technoscience, sometimes in prescient anticipation of its slow and forgetful “discoveries,” perhaps often also superior and in certain ways richer than it, one has to face the fact that Western technoscience is hegemonic in political and regulatory terms and that, under such hegemony, the conditions under which such techniques will be retrieved from that archive and redeployed are likely to be determined to a significant extent by the criteria determined by that paradigm. Despite Michaux's indifference to indigenous experience and history, I nevertheless take the view that his drug works also contain valuable resources with which we can reconceptualize the politics of psychedelics, including in ways which will ultimately favor well-founded demands for “psychedelic justice” (Cavnar and Labate, 2021), among them for the recognition—in a substantial sense exceeding mere rhetoric and virtue-signaling—of indigenous expertise, stewardship, and tradition.^22^ When reading, one need not bow to pressure to accept or reject everything en bloc: one can analyze, differentiate, and reassemble—indeed, this is what it means to read critically.

## 4. Michaux's mystical naturalism

The dominance of the psychotomimetic paradigm of psychedelic efficacy in the biomedical science of Michaux's day probably contributed to the difficulty of some of his early experiences and, in turn, to his hostile early judgments. Yet, as they develop, his drug writings reveal that this mindset changed gradually, with repeated practicing of the psychedelic experience, culminating in a stark divide between the first and second books.^23^ Despite attempting to stick with his initial skepticism and maintain an agonistic, distanced, scientific, and observational relationship to the drug (Michaux, 2001; p. 847), in the second book he eventually reports a full-blown mystico-religious experience, in block capitals: “I HAVE SEEN THE THOUSANDS OF GODS” (Michaux, 2001; p. 852). He also claims to experience being traversed by, in a sense, of one substance with, a wave of energy he calls “the furrow” [*le sillon*], “Furrow without beginning or end […], which I would say comes from one side of the world, traversing me as it moves to the other” (Michaux, 2001; p. 626). As his practicing of the drug proceeds, this initially harrowing experience is acknowledged in some sense to be the revelation of a valid metaphysical intuition about the universe and his set changes from resistance to acceptance: “I stopped struggling, I let myself be traversed by the fluid which, entering by way of the furrow, seemed to come from the end of the world” (Michaux, 2001; p. 648–9). Unsurprising too that in the second book (Michaux, 1957), he draws on Christian mystics, including Catherine of Siena (Michaux, 2001; p. 914); in the third (Michaux, 1961), he recounts hallucinating snippets of “Trois Petites Liturgies de la présence divine” (1944), a cantata by the devout Olivier Messiaen. However, these mystico-religious experiences do not challenge Michaux's implicit commitment to philosophical naturalism and strong physicalism, or in other words, the belief that physics offers a complete description of causality, that the universe is as the natural sciences describe it, that some physical entities lack mental properties, and those physical entities with mental characteristics evolved from physical entities with no mental characteristics [for a fuller account of these related positions see Angel (2002); p. 317-8]. In the “Addenda” to the first book, published only in the 1972 edition, after the completion of the other texts of the cycle, he testifies to what amounts to lasting personality change over the intervening years, under the influence of psychedelics, yet this too is entirely intelligible within the frame of naturalism: “Strange! I have become active. Attentive to what is happening—in and of itself—without trying to deform it or imagine it differently to make it more interesting to me” (Michaux, 2001; p. 770).

Regarding the epistemological reliability of the mystical experiences some users encounter under psychedelics, in particular the status of visions of what is sometimes called “other entities,” there are two opposing extremes in the current literature, exemplified in recent scholarship by Chris Letheby's plea for a “natural philosophy” of psychedelics (Letheby, 2021; p. 8), one which is compatible with naturalism and physicalism, on the one hand, and Peter Sjöstedt-Hughes's conviction that mystical experiences under psychedelics constitute evidence for panpsychism (Sjöstedt-Hughes, 2021), on the other. Michaux's work demonstrates *both* a strong mystical impulse *and* a strong commitment to naturalism and physicalism: not only is there no sense of dissonance between mysticism and naturalism but in many ways, Michaux's work succeeds in integrating them, such that his position might be characterized as “mystical naturalism” (Angel, 2002), or a “mystic materialism” of the type Huxley and Leary espoused, perhaps even one anticipating the “biomysticism in awe of life itself” which Nicolas Langlitz has seen gradually emerging from the intersection between neuroscience and psychedelics (Langlitz, 2012; p. 255).

Michaux's mysticism nevertheless requires careful reading to discern its fidelity to naturalism and physicalism: sometimes he comes quite close to suggesting that this experience might be evidence of the real existence of other entities. As Blanchot noted perceptively: “Someone we have every reason to believe has met the gods. Unique revelation. But do we gather around this encounter? Do we forsake our occupations, our thoughts, to consider so significant an affirmation? Not in the slightest. Even Michaux's admirers speak of the incident without emotion. For a start, I note this indifference.” (Blanchot, 1966; p. 83). Michaux is, in a sense, convinced by his mystical experiences but only quality experience in a restricted, implicitly subjective sense of the term compatible with naturalism and physicalism. The mystical visions he sometimes experienced under psychedelics do not cause him to question this framework, though at times he comes quite close to doing so.

As Michaux reflects on his experience of mescaline, he traverses many different ways of envisioning the drug's effects, of which mystical visions are just one: from mystical encounters with other entities and cosmic energies, he passes through figurations of his serfdom to the drug to become the object of its feminine seduction and be queerly penetrated by it. Passing through these different figurative plateaux, Michaux gradually embraces the belief that the drug reveals a power within himself that is also other than himself, which I have termed elsewhere the “autoheteronomous”: “a reserve within me, a zone x, an zone in waiting of which I had had no knowledge,” “both a third party and yet purely myself” (Michaux, 2001; p. 773; Davis, 2022; 679). From the perspective of my reading of Michaux and the program I glean from him for the psychedelic humanities, what matters most is the potential for a creative individual, social and political transformation in the intelligent and skilled use of these substances within the frame of scientific naturalism and physicalism: the psychedelic humanities must, I would argue, chart a scientifically enlightened path—but rather than the sobering prospect of a natural philosophy of psychedelics, Michaux's work suggests that a more promising paradigm, which better captures the force of psychedelic experience, might be mystical naturalism.

## 5. Conclusion: the concept of psychedelics as anthropotechnics and a note on Michaux's “program”

This article has argued that the drug works by Henri Michaux make a substantial contribution to the cultural understanding of psychedelics in three areas: (1) the role of psychedelics in enhancing “creativity”; (2) conceptualization of the politics of psychedelics; and (3) mystical naturalism. In this way, I have gleaned from the treasury that Michaux's work constitutes a “program” for research in the psychedelic humanities. I must emphasize, in part, because this became a source of contention during the review process: a program, not the program. In siding so resolutely with Western technoscience and biomedicine, in its preference for synthesized laboratory chemicals over plant medicines and its relative lack of interest in indigenous cultural practices, Michaux's program is certainly out of step with much research in the field today. In reconstructing Michaux's engagement with drugs as a “program,” I am not proposing that any other approaches thereby be displaced or invalidated. At the same time, Michaux's vision has an integrity and honesty of its own which should not quickly be disparaged and, limited though it is in other respects, he assuredly does have substantial contributions to make to contemporary debate in the three areas I have outlined.

Finally, a note on the method. Implicitly, my analysis has envisaged Michaux's work in terms of philosopher Peter Sloterdijk's conception of “anthropotechnical practicing” (Roney and Rossi, 2021), whereby psychedelics are anthropotechnics (tools, techniques, or technologies for the modification of the human), and this way of conceptualizing psychedelics is, I believe, a valuable—indeed, perhaps, foundational—theoretical framework for research in the psychedelic humanities. For Sloterdijk, humanity is a self-enhancing species: we deploy anthropotechnical tools, learn from the experience, refine, and repeat in an elevating cycle of practicing to develop performance and yield. Some critics have lamented Michaux's repetitiveness in the drug works (Bowie, 1973; p. 151; Parish, 2007; p. 74). However, their somewhat repetitive character makes more sense when they are envisaged as the record of a program for self-enhancement by repeated practicing with psychedelic anthropotechnics. For Sloterdijk, the anthropotechnical instruments of education in the humanities, from their emergence in the 19th century, were books. For the psychedelic humanities, psychedelics assembled with other techniques (including books and other cultural objects—these are not to be supplanted, contrary to Leary's suggestion) perform a similar educative function, yielding individual and social transformation. A “program” is also a script, a set of choices and outcomes that can in turn be fed back into new experiments with new assemblages of psychedelics and other anthropotechnics, as John Lilly and Erik Davis, commenting on Lilly, have envisaged (Lilly, 1968; Davis, 2019; p. 32). Unfortunately, because of the extent and difficulty of Michaux's own works and for contingent reasons of translation, the insights they contain have too long remained the preserve of a happy few and so not been available for such redeployment. Although the account I have given here is necessarily selective in its coverage, I have tried to focus on three particular areas in which Michaux's work has something substantial to contribute to ongoing conversations today, while also acknowledging its limitations.^24^

## Data availability statement

The original contributions presented in the study are included in the article/supplementary material, further inquiries can be directed to the corresponding author.

## Author contributions

The author confirms being the sole contributor of this work and has approved it for publication.

## Footnotes

^1^This description of Michaux's work has proven to be more controversial than I had initially anticipated, factually and politically. In the confines of this article, I am not in a position to independently substantiate it, since to do so would require a comprehensive treatment of major mescaline-influenced creative work and wider cultural practice during the period; however, the claim is made within precisely such an extensive cultural history of mescaline (Jay, 2019) and the purpose of citing it here is to give some initial indication of the significance of Michaux's work and to invite the reader to devote some of their time to engaging with my analysis. Nothing turns argumentatively, for me, on Michaux's being preeminent in the way Jay's comment implies. Politically speaking, the claim could be felt to ignore, or as the more dramatic verb would have it, to *erase*, the very significant work of the Native American Church in using peyote over the century for “postcolonial healing” (Calabrese, 2013). Without wishing to downplay the significance of the NAC's work, I would suggest that its therapeutic ritual use of peyote is not primarily creative in the sense in which artists and psychologists understand this term, in particular, in so far as it rests on a “core therapeutic emplotment” within a “very uniform” ceremonial structure (Calabrese, 2013; p. 124). Controversially, for the psychedelic humanities today, and surprisingly given that he had traveled extensively in Central and South America earlier in his life, Michaux was not interested in peyote or in indigenous uses of that mescaline-containing cactus; his interest was in just one of its spectrum of alkaloids in one of its laboratory-synthesized forms, mescaline hydrochloride. I discuss the political significance of this choice in §3.

^2^Underappreciated but not unread: early book-length studies of Michaux's drug writing by psychiatrists and doctors (Ajuriaguerra and Jaeggi, 1963; Loras, 1967) approached it largely in terms of the psychotomimetic paradigm. The literary critic and philosopher Maurice Blanchot is dismissive of such readings in a characteristically perceptive article first published in 1958 (Blanchot, 1966), perforce covering only the first two books: “It would be more instructive to speak of a simple attitude such as impatience, which also changes the experience of time.” (86) This and all subsequent translations from French are my own. Prompted by the title of the second book, *L'Infini turbulent* (Michaux, 1957), Blanchot suggests that mescaline allows Michaux an unsettling experience of the infinite (81) and enables him to sketch “a new form of literature” (87). A notable later monograph (Brun, 1999) adopts a psychoanalytic approach, likening Michaux's mescaline writings to Freud's 1884 essay on cocaine (42) and argues that psychedelics enabled Michaux to escape from feelings of being imprisoned in his body which he had experienced since childhood (65). Anne Brun also suggests that the abundance of onomatopoeia is an attempt to render a language of the body reminiscent of the moment when the infant discovers language (93), an argument clearly indebted to that advanced about the reemergence of the ‘semiotic' in poetic language (Kristeva, 1974). Literary critic Max Milner devotes a detailed and astute chapter to Michaux's drug writings in his book on drugs and the imagination (Milner, 2000), underlining Michaux's “ascetic” (374) and “agonistic” (403) relationship to the substances. Many monographs on Michaux's work as a whole mention the mescaline decade somewhat disapprovingly (e.g., Bowie, 1973; Parish, 2007). Peter Broome suggested that mescaline provided Michaux with “an incredible new projector for his inner cinema” (Broome, 1977; p. 89). The notes and other scholarly apparatus in the Pléiade edition of Michaux's works (Michaux, 2001, 2004) are an extremely rich resource, extensively exploited in the present article. Devenot et al. (2022) suggested that even to speak of a “psychedelic renaissance” is to fall for a confidence trick on the part of pharmaceutical entrepreneurs and “psychedelic pundits” keen to monetize psychedelics while erasing indigenous cultures' stewardship of plant medicines and maintaining prohibition.

^3^The 1972 French edition of *Misérable Miracle* was much more enthusiastic about mescaline than the 1956 first edition, which was translated into English (Michaux, 1963a), thanks to the addition of “Addenda” much more in keeping with the more mystical and positive appreciation of the drug in *L'Infini turbulent* (Michaux, 1957).

^4^All page references to Michaux's published work in this article are given to the scholarly editions of reference, volumes 2 and 3 of Michaux's Œ*uvres complètes* in the Bibliothèque de la Pléiade (Michaux, 2001, 2004), rather than to the various original editions. Two editions of *L'Infini turbulent* appeared during Michaux's lifetime: the original edition of 1957 and a slightly revised edition published in 1964. The discussion about the *Bardo Thödol* occurs in both editions.

^5^I am grateful to one of the reviewers of this article for introducing me to McClure's remarkable body of work and, in particular, the *Meat Science* essays. McClure was inspired to write his *Drug Notes* after reading a translation of an excerpt from *Misérable Miracle* in the *Evergreen Review* (McClure, 1993; p. ix).

^6^Rouhier's magnificent dedication reads in full: “I DEDICATE THIS BOOK TO THE SPIRIT OF CUAUHTEMOC, ‘THE EAGLE WHO DESCENDED', HIGH PRIEST OF THE AZTEC RELIGION, CUTTER OF THE PEYOTE AND LAST EMPEROR OF THE ANAHUAC, WHOM THE CHRISTIAN CONQUISTADORS, HUNGRY FOR GOLD, TORTURED BY FIRE AND HANGED ON THE TWENTY-FIRST OF AUGUST IN THE YEAR OF GRACE M D XX II” (Rouhier, 1927; p. iii, capitalization in original).

^7^Before 1970, the principal domestic drug control legislation in France was the 1916 law (Yvorel, 2012) and in 1957, mescaline was added by administrative decision to the list of substances under the purview of that law. Nevertheless, two peculiarities of 20th-century French drug control law deserve mention: first, that between the passing of the 1916 law and the tightening of domestic legislation in 1970, neither the mere possession of controlled substances in private without intent to sell them on nor their use in private were prohibited by law (Marchant, 2018; Black, 2022), even though police and prosecutors would often behave as though they were (Retaillaud-Bajac, 2009; p. 231-271). Second, to this day, French law continues to place highly unusual emphasis on punishing what is characterized as proselytising for drugs in Article L3421-4 of the Code de la santé (Légifrance, 2007): in effect, offering any representation that is not harm-focused. This internationally unusual restriction continues to exert an inhibiting effect on cultural production, grown-up public debate, and, in turn, on the allocation of funding for research on psychedelics (Chayet, 2020). In Michaux's case, the ambivalence characterizing his published works on drugs makes it difficult to construe them straightforwardly as proselytism for drugs and various harms are mentioned, although a plausible argument could certainly have been made in that direction and might well have been, had he been less well connected and more widely read.

^8^Michaux had emigrated to Paris from his native Belgium in the 1920s and gone on to travel widely in Central and South America, the Middle East, and East Asia, before settling for good in Paris (Ouvry-Vial, 1989; Martin, 2003). He had lived alone but for a housekeeper since the death of his wife in a domestic accident in 1948 (Martin, 2003; p. 441-2).

^9^I shall return to reflect on the significance of this particular trip in §2, below. When he felt things were getting out of hand, Michaux also sometimes resorted variously to the following real and imagined antagonists: sugar, oranges, benzodiazepine chlordiazepoxide (Librium), nicotinamide (vitamin B3), Véricardine (a heart medication containing phenobarbital), and even the stimulant camphor (Solucamphre) (Halpern, 1998; p. 106).

^10^In this section, I generally place “creativity” within scare quotes because (a) it is notoriously difficult to define and measure psychologically (Said-Metwaly et al., 2017); (b) it has largely displaced the more venerable and broader concept of the esthetic, which I feel would in many ways be preferable; (c) despite the near-universal scientific and popular consensus that it is a good thing, it has a sinister history of entanglement with the politics of corporate “innovation”and technocratic rule, reflected in the favor it finds today among microdosing Silicon Valley tech workers who design and refine the digital instruments of global governance.

^11^Their explicit technical-motivational conception of psychedelically enhanced creativity in terms of improved problem-solving ability and greater willingness to succeed was prominent in pre-prohibition scientific research and is neatly encapsulated in the title of one influential book-length study: *LSD: The Problem-Solving Psychedelic* (Stafford and Golightly, 1967). Harman et al. (1966) is also a foundational point of reference in this book, which collates the accounts of problem-solving scattered through a biomedical literature of the day which was more directly orientated toward therapeutic use. The authors envisage psychedelics beyond their medical use as aiding “the alleviation of those countless problems by which man [*sic*] is beset” (Stafford and Golightly, 1967; p. 30), or in other words for the general enhancement of human activity by solving problems in all manner of areas: “business, pleasure, sickness, health, birth, death, ad infinitum” (30).

^12^The “primary active agent” in the experiments that were written up was 200 mg of mescaline sulfate (Harman et al., 1966; p. 216). However, in common with a certain amount of other early research (for instance, Martin, 1962), the psychedelic was co-administered in this study with a stimulant, in this case, methedrine (methamphetamine) but with scant discussion of the likely impact of the second drug. One of the co-authors, psychologist James Fadiman, later gave an evasively euphemistic account of the second part of this drug combination: “The dose was 50 micrograms of LSD, preceded by *energizers*.” (Fadiman, 2011; p. 168, emphasis added), the reference to LSD probably being to the informal preliminary sessions before those written up. While it is hardly surprising that biomedical researchers tentatively resuming work on psychedelics in the renaissance should have steered well clear of methamphetamine, now the most abhorred of substances (Parsons, 2014), it is remarkable that the impact of co-administration of psychedelics with stimulants in this and other early research has yet to be discussed or replicated.

^13^Given that terms used to name elements within this creativity cluster vary between studies, I note that the perceptual and cognitive dimensions might together have been described as “aesthetic” aptitudes, according to a long-established use of this term in philosophy which has nevertheless fallen from favor in psychology. Although this “aesthetic” dimension was not addressed in such terms in the discussion of their findings by the study's authors, it was alluded to briefly (as “aesthetic sensibility”) in the somewhat amorphous working definition of creativity deployed in the study from Carl Rogers (216): by “aesthetic”, I refer both to the perceptual and the cognitive dimensions involved, for example, in the capacity to see pattern *as* pattern, or “visualize” the solution (Harman et al., 1966; p. 224)—as well as that which pertains to the beauty of its form, for example, in the “elegance” of a “solution,” a way of speaking common in mathematics and technology, which also suggests economical use of symbolic or other materials. The relevance of this point will become clear in §3. Two recent studies (Kuypers et al., 2016; Mason et al., 2021) try to focus more precisely on two dimensions widely thought to be important dimensions of creative thinking: divergent and convergent thinking. The first (Kuypers et al., 2016) found an increase in divergent thinking during the acute phase following ayahuasca administration; the second (Mason et al., 2021) did not find the same effect with psilocybin but did find that divergent thinking and creative cognition had increased after the acute effects had worn off, 7 days after administration.

^14^There is one obvious, principled but pedestrian reason why this is the case: a single artist's experience can count with no more weight than any other participant in the statistical analysis of experimental results. However, in the design of studies to investigate “creativity” and its psychedelic enhancement, working definitions of “creativity” must to some extent be abstracted from a cultural conversation that remains very confused about what that elusive quality might be but in which, nevertheless, art and artists have since time immemorial generally been recognized to have a certain kind of proficiency or prominence. Appealing to a standard-issue conception of the scientific method as an excuse not to take any account of the collective wisdom reflected in that social and historical fact is itself a methodological decision with consequences, not least in that not only the results but also the working assumptions of such narrowly conceived research on “creativity” also feed back into the culture in a way which further flattens it to fit the thin template of techno-scientific rationalism. One task of the psychedelic humanities is to arrest this downward spiral.

^15^The resulting layout of the page bears a passing resemblance to some medieval manuscripts which incorporate marginal annotations and glosses, although the italicized marginalia in Michaux's case generally sit in a more conflictual, unstable, and less harmonious relationship to the adjacent column.

^16^The innovative formal doubling of the written text into text and paratext is by far the most significant of these proliferations, given that in some of his other work beyond the drug series, he also juxtaposes writing and visual art. See Parish (2007).

^17^Mouchard (1979; p. 168-9) noted that the title of the first book in the drug series, *Misérable Miracle*, contains a phonetic reflection of its author's surname in the repeated first syllable of each word; given Michaux's interest in onomastics elsewhere, this is a pertinent observation. Here, the double m, in “immenses,” refers us back to the title and the author's surname even while it serves as the occasion of Michaux's first glimpse of the drug's enhancement of his capacity for self-suggestion. This line of interpretation could be pushed further: given the dramatic reversal in affective attitude between the first and second books (see §4, below), the move from the “me, me” of *Misérable Miracle* to the “it” of *L'Infini turbulent* might be understood as the expression, drawing on the rudiments of a foreign language with which Michaux was passingly familiar, of the transition from an agonistic ego-focused relationship to the drug to one more accepting of its “autoheteronomous” activity.

^18^The influence of Michaux on Deleuze and Guattari has been noted by Raymond Bellour (Michaux, 1998; p. lix-lxi), who remarks on the long quotation from one of the drug books (Michaux, 1961) in the early pages of *Anti-Oedipus* (Deleuze and Guattari, 1984 [1972]; p. 6-7). Although talk of “molecules” evidently abounds in science and the wider culture, it may well be that “the molecular,” in the precise sense it acquires politically in this body of work, was also drawn from Michaux and from the first study of his drug writing, in which the “the molecular swarming of elements” and “the as it were molecular disturbance of the constituents of thought” are particular points of emphasis (Ajuriaguerra and Jaeggi, 1963; p. 12, 44). The genealogy of Rancière's political thought presented in outline here may surprise some: there are, of course, other important elements he brings to—and assembles with—the “molecular” vision of politics outlined by Guattari and Deleuze but, in my considered view, there is no doubting the continuity of this line of thinking.

^19^I do not mean to imply that psychedelics have never been used by authoritarian political regimes or for abusive purposes by repressive state agencies working within ostensibly democratic countries. Mescaline, among other drugs, was used in experiments on prisoners at Dachau by Dr. Kurt Plötner as part of the Nazis' search for a truth serum to facilitate interrogation. In parallel, the Truth Drug Committee of the US Office of Strategic Services, the forerunner of the CIA, trialed mescaline in 1942 (Jay, 2019; p. 185). When the war ended, Plötner was recruited by the Americans and went on to work in Project Bluebird, later absorbed into the MKUltra project. Bluebird and MKUltra sought to deploy psychedelics and other substances and techniques for mind control and behavior modification and included experimentation on subjects who had not given their consent, in violation of the Nuremburg Code. For the purposes of my “redundancy thesis,” the key conclusion to be salvaged from this sickening history is that even the CIA concluded that psychedelics were unsuitable for their purposes because they were far too unpredictable in their effects.

^20^Further discussion of this matter would take me too far away from Michaux. It is the point of departure for my forthcoming book on the politics of psychedelics.

^21^I elaborate on the argument presented in this section in my forthcoming book on the politics of psychedelics. In so far as neoliberal capitalism already relies on bureaucracies of ranking and is committed to self-optimization on the part of its subjects, it might be able to make especially effective use of this superlative or “maximomaniacal” propensity in psychedelic experience.

^22^I explain in more detail how in my forthcoming book on the politics of psychedelics.

^23^In the second edition of Michaux (1956), published by Gallimard in 1972, the sceptical and pessimistic verdict on mescaline delivered in the main body of the text is flatly contradicted by a series of appendices revealingly entitled “Addenda,” in which Michaux presents a much more positive view of the psychedelic, inflected by his subsequent practicing with the drug.

^24^Full discussion of the film, in conjunction with the drawings, is reserved for a future article.
